# Evolution of cardiac and renal impairment detected by high-field cardiovascular magnetic resonance in mice with renal artery stenosis

**DOI:** 10.1186/1532-429X-15-98

**Published:** 2013-10-26

**Authors:** Behzad Ebrahimi, John A Crane, Bruce E Knudsen, Slobodan I Macura, Joseph P Grande, Lilach O Lerman

**Affiliations:** 1 Division of Nephrology and Hypertension, Mayo Clinic, Rochester, Minnesota, USA; 2 Department of Laboratory Medicine and Pathology, Mayo Clinic, Rochester, Minnesota, USA; 3 Department of Biochemistry and Molecular Biology, Mayo Clinic, Rochester, Minnesota, USA

**Keywords:** Renal artery stenosis, LV dysfunction, Ultra-high field MRI, BOLD, ASL, Hypoxia

## Abstract

**Background:**

Renal artery stenosis (RAS) promotes hypertension and cardiac dysfunction. The 2-kidney, 1-clip mouse model in many ways resembles RAS in humans and is amenable for genetic manipulation, but difficult to evaluate noninvasively. We hypothesized that cardiovascular magnetic resonance (CMR) is capable of detecting progressive cardiac and renal dysfunction in mice with RAS and monitoring the progression of the disease longitudinally.

**Methods:**

RAS was induced at baseline in eighteen mice by constricting the renal artery. Nine additional animals served as normal controls. CMR scans (16.4 T) were performed in all mice one week before and 2 and 4 weeks after baseline. Renal volumes and hemodynamics were assessed using 3D fast imaging with steady-state precession and arterial spin labelling, and cardiac function using CMR cine. Renal hypoxia was investigated using blood oxygen-level dependent (BOLD) MR.

**Results:**

Two weeks after surgery, mean arterial pressure was elevated in RAS mice. The stenotic kidney (STK) showed atrophy, while the contra-lateral kidney (CLK) showed hypertrophy. Renal blood flow (RBF) and cortical oxygenation level declined in the STK but remained unchanged in CLK. Moreover, cardiac end-diastolic and stroke volumes decreased and myocardial mass increased. At 4 weeks, STK RBF remained declined and the STK cortex and medulla showed development of hypoxia. Additionally, BOLD detected a mild hypoxia in CLK cortex. Cardiac end-diastolic and stroke volumes remained reduced and left ventricular hypertrophy worsened. Left ventricular filling velocities (E/A) indicated progression of cardiac dysfunction towards restrictive filling.

**Conclusions:**

CMR detected longitudinal progression of cardiac and renal dysfunction in 2K, 1C mice. These observations support the use of high-field CMR to obtain useful information regarding chronic cardiac and renal dysfunction in small animals.

## Background

Cardiovascular disease (CVD) associated with renovascular disease is a major cause of morbidity and mortality among patients with chronic kidney disease (CKD) [1-3]. Renal artery stenosis (RAS) is associated with renin-angiotensin system driven hypertension, which initiates a cascade of biological events including mechanical signalling, leading to left ventricular (LV) hypertrophy (LVH) and dysfunction [4,5]. In addition to traditional causes of CVD such as hypertension, the progression of CVD may be related to the duration and severity of non-traditional risk factors, such as renal hypoxia and oxidative stress [6,7]. This has attracted a great deal of interest to study the longitudinal association between RAS, renovascular hypertension and CVD, such as LV dysfunction [8]. The two-kidney, one-clip (2K, 1C) model is often used to induce renovascular hypertension in small animals [9,10]. While many studies were performed in rats, due to the larger size of their renal artery, which facilitates the surgical procedure, in recent years this model has been developed in mice, mainly due to the possibility of combining this procedure with genetic manipulations [11-13].

Several imaging modalities can provide important diagnostic and prognostic insights into cardiac and renal dysfunction in RAS [14]. In the kidney, renal Doppler ultrasound is useful to detect and assess renal blood flow (RBF) [15,16]. Similarly, cardiovascular magnetic resonance (CMR) is able to evaluate renal volume and hemodynamics [17-19]. Moreover, blood oxygen-level dependent (BOLD) MRI has the capacity to non-invasively measure renal cortical and medullary oxygenation level, while the only current alternative requires invasive microelectrodes [20,21]. Renal hypoxia is an important marker of progressive kidney dysfunction and contributes to development of hypertension [22].

Echocardiography has been extensively used for quantitative LV characterization [23] using important functional parameters such as LV end-diastolic and end-systolic volumes, ejection fraction, myocardial muscle mass, and early to late filling velocity ratio (E/A), an index of diastolic function. Similarly, CMR is capable of assessing cardiac function and detecting myocardial remodelling [24]. However, application of cardiac MRI in mice remains limited mainly due to challenges associated with the temporal and spatial constrains imposed on clinical CMR systems by the small size and high heart rate in cardiac imaging of small animals [25].

Ultra-high field (UHF) MRI has surpassed some limitations of small animal CMR imaging and improved cardiac imaging in mice [26]. The modality is gaining popularity owing to its improved sensitivity. Yet, the capability of UHF-CMR to detect cardiac and renal alterations in vivo in mice with RAS and follow them longitudinally has not been explored.

The aim of this study was to determine if UHF-CMR can non-invasively quantify crucial parameters of cardio-renal dysfunction and the progression of LV dysfunction secondary to RAS in 2K, 1C mice model.

## Methods

All animal procedures were performed in accordance with the National Institutes of Health Guide for the Care and Use of Laboratory Animals, and approved by the Mayo Clinic Institutional Animal Care and Use Committee.

The study was performed on 27 male 129S1 mice (Jackson Lab, Bar Harbor, ME). Animals arrived to the lab at 6 weeks of age, were acclimatized to blood pressure measurements, and underwent a baseline CMR scan a week later. Then mice were randomly divided into two groups. RAS was induced in the first group (n = 18) one week after the baseline scan by surgical placement of an arterial cuff. The second group (n = 9) served as the control Normal. All the animals underwent additional scanning 2 and 4 weeks later.

### Surgical procedure

Animals in the RAS group were anesthetized with Isoflurane (1.5-2.0%). The right lateral area between the margin of the rib cage and thigh was shaved and scrubbed with povidone-iodine solution. The right kidney was exposed by a flank incision (~1 cm) and reflected midline. The right renal artery was then bluntly dissected from the renal vein between the renal bifurcation and the bifurcation of the testicular artery. A 0.2 mm ID × 0.5 mm length cuff (Polyetrafluoroethylene, Braintree Scientific, Braintree, MA) was cut longitudinally, placed around the renal artery, and secured with 10-0 nylon suture. Kidney was returned to its native position and the incisions were sutured as described [9,27].

Mice were recovered on a warm (37°C) pad until ambulatory, and then returned to normal housing with ad libitum access to standard chow and water. Blood pressure and heart rate were measured before, at 2 weeks and at 4 weeks after baseline by tail-cuff, using a XBP1000 system (Kent Scientific, Torrington, CT). Animals were euthanized within a day after the 4-weeks scan. The kidneys and the heart were harvested and fixed for immunohistochemical staining.

### Imaging protocol

Imaging was performed on an Avance DRX 700WB (Bruker-BioSpin, Billerica, MA) spectrometer with a vertical 16.4 T wide bore magnet equipped with mini imaging accessories. A 38 mm volume RF coil was used for the kidney and cardiac imaging. Mice were mounted vertically along the magnetic field in a home-made animal holder. Cardiac and respiratory monitoring and gating were performed using the Control/Gating Module of Model 1030 Monitoring and Gating System (SA Instruments Inc., Stony Brook, NY). Body temperature was measured through a rectal sensor, using temperature module and maintained at 37°C throughout the course of experiment. Respiration was measured through a balloon sensor attached to Respiration Module and monitored using the software provided by SA Instruments. Using 3 planes (sagittal, coronal and axial), scout images were collected to locate the heart and kidneys and prescribe the geometry for anatomical and functional imaging.

Renal perfusion and volume were measured in all animals. Renal blood oxygenation level and cardiac function were measured in all control animals (n = 9) as well as in 12 of the 18 RAS animals.

The kidney volumes were estimated from images collected using respiratory-gated 3D Fast Imaging with Steady Precession (3D-FISP) [28] (TR/TE 14/2.1 ms, matrix 512 × 256 × 128, FOV 5.12 × 2.56 × 1.28 cm, flip angle 20°). Images were collected axially and reconstructed into 3-dimensional volumetric images.

Perfusion was measured by arterial spin labelling (ASL) method [18] using a flow-sensitive Alternating Inversion Recovery (FAIR) sequence, with Rapid Acquisition with Relaxation Enhancement (RARE) (1-2 axial slices per kidney, with TR/TE 10000/5 ms, RARE factor 72, matrix 128 × 128, FOV 4 × 4 cm, slice thickness 1 mm). A total of 22 images with inversion times of 40 to 2300 ms were collected.

BOLD CMR was applied using respiratory-gated conventional 2-dimensional Multi-Gradient Echo (MGE) sequence [29] (axial images with 8 echoes (ΔTE = 3 ms) with TR/TE 200 ms/3.5-24.5 ms, matrix 256×256, FOV 4 × 4 cm, slice thickness 1 mm, flip angle 25°). Global shimming was performed for all kidneys. In cases of repeated failure of the shimming algorithm to converge, shimming was performed locally.

In some animals (n = 12) BOLD CMR images were also collected using 3-dimensional MGE to investigate the feasibility of reducing the effect of the external field inhomogeneity and susceptibility artefact [30]. The same FOV as that in conventional BOLD was covered by 16 slices, raising the matrix size to 256 × 256 × 16. Then, magnitude images of all 16 slices were added to generate a single 1 mm reconstructed image. In improved BOLD, all other parameters were kept the same as for the conventional BOLD sequence.

The cardiac cine images were acquired using IntraGateFast Low-Angle Shot (ig-FLASH) sequence [31] (4-6 short axis slices, TR/TE 3.5/1.45 ms, repetition 100, matrix 256 × 256, FOV 3.2 × 3.2 cm, slice thickness 1 mm).

### Data analyses

Kidney volumes were calculated from 3D-FISP data using Analyze™ (Biomedical Imaging Resource, Mayo Clinic, MN). Corticomedullary borders were evident in the MR images and used to differentiate cortical and medullary regions of interest (ROI).

ASL Tool Module (Bruker-ParaVision) was used to calculate cortical and medullary perfusion in both the stenotic and contra-lateral kidneys. Blood flow for each compartment was calculated by multiplying its perfusion and corresponding volume, and RBF calculated as their sum.

To calculate the BOLD index, R_2_* (1/T2*), we took advantage of the high contrast of cortex and medulla in ASL images to differentiate the two compartments. ROIs were drawn on ASL images and then transferred to all BOLD images corresponding to the 8 echo times. Voxel-based R_2_* values were calculated by fitting the decaying signal of each ROI to a mono-exponential curve vs. echo times, and cortical and medullary R_2_* values by averaging the R_2_* values for all voxels within the corresponding ROIs.

Cardiac function was calculated from cine images, reconstructed by Paravision-IntraGate Module. LV myocardial volume was estimated by drawing ROIs around the outer and inner segments of the myocardium, calculating the difference, and multiplying by the slice thickness (Simpson’s rule) [32]. The stroke volume was calculated as the difference of volumes at the end diastolic and systolic phases. The ejection fraction was assessed by normalizing the stroke volume to the end-diastolic volume, and cardiac output by multiplication of the stroke volume and heart rate. The E/A ratios were calculated from the slopes of the volume-time curve observed at early (E) and late (A) diastole, utilizing cardiac images reconstructed at rates of 10, 25 and 40 phases per cardiac cycle using IntraGate module [33]. The data analysis was performed using Analyze™ and MATLAB® (MathWork, Natick, MA, USA).

### Staining

HIF-1α and trichrome were stained on 5-μm axial slides of tissue using Rabbit polyclonal antigen (Lifespan Biosciences Inc. Seattle, WA) with working dilution of 1:100. EDTA was used for antigen retrieval.

### Statistical analysis

Minimum sample size was calculated using power analysis for the power value of 0.8. Non-parametric Wilcoxon signed-rank test was used for comparisons within groups and Mann-Whitney for comparison among groups. For p values smaller than 0.05, differences were considered significant.

## Results

Blood pressure measurements at 2-weeks demonstrated development of renovascular hypertension in RAS. Mean, systolic, and diastolic blood pressures were significantly increased in RAS and remained elevated throughout the study, while control blood pressure remained unchanged(Table 1). Two weeks after the surgery, RAS animals lost 1.7 ± 0.6 g of their body weight, but regained it by the fourth week. The control group gained weight during the course of the study.

**Table 1:** Blood pressure, renal volumes and hemodynamic parameters in renal artery stenosis (RAS) or control mice

	**Baseline**	**2 weeks**	**4 weeks**
Blood pressure (mmHg)			
RAS MAP	80 ± 1	112 ± 5*^$#^	118 ± 5*^$#^
Diastolic	72 ± 2	99 ± 4*^$#^	106 ± 5*^$#^
Systolic	98 ± 3	138 ± 6*^$#^	144 ± 6*^$#^
Control MAP	83 ± 1	78 ± 1	83 ± 3
Diastolic	76 ± 2	69 ± 2	76 ± 4
Systolic	97 ± 2	96 ± 4	99 ± 2
Body weight (g)			
RAS	20.7 ± 0.7	19.0 ± 1.2*^$#^	21.1 ± 1.1^#^
Control	21.0 ± 0.9	23.2 ± 0.7*	23.8 ± 0.6*
Volume (mm^3^)			
Stenotic Kidney			
Cortex	141 ± 8	86 ± 8*^$#^	91 ± 8*^$#^
Medulla	47 ± 2	23 ± 4*^$#^	18 ± 3*^$#^
Contralateral Kidney			
Cortex	140 ± 7	158 ± 7	179 ± 8*^$#^
Medulla	46 ± 3	42 ± 5	38 ± 3*^#^
Control			
Cortex	135 ± 5	140 ± 3	146 ± 6
Medulla	44 ± 3	42 ± 2	51 ± 3
Flow (μl/min)			
Stenotic Kidney			
Cortex	613 ± 48	224 ± 35*^$#^	194 ± 35*^$#^
Medulla	75 ± 5	24 ± 6*^$#^	18.0 ± 4*^$#^
RBF	688 ± 46	248 ± 40*^$#^	212 ± 39*^$#^
Contralateral Kidney			
Cortex	643 ± 50	650 ± 35	728 ± 52
Medulla	73 ± 4	62 ± 10	55 ± 8^#^
RBF	716 ± 49	712 ± 41	783 ± 56
Control			
Cortex	611 ± 18	680 ± 21	650 ± 35
Medulla	77 ± 5	74 ± 5	83 ± 6
RBF	688 ± 18	754 ± 24*	733 ± 40
Perfusion (ml/100 g/min)			
Stenotic Kidney			
Cortex	434 ± 22	239 ± 27*^$#^	194 ± 30*^$#^
Medulla	168 ± 18	90 ± 11*^$#^	85 ± 15*^$#^
Contralateral Kidney			
Cortex	458 ± 22	416 ± 16^#^	409 ± 24
Medulla	165 ± 13	139 ± 13^#^	134 ± 17
Control			
Cortex	455 ± 12	487 ± 10	443 ± 14^†^
Medulla	173 ± 5	176 ± 10	163 ± 6

*P < 0.05 vs. Baseline RAS, ^$^P < 0.05 vs. Baseline Control, ^†^P < 0.05 vs. 2 weeks, ^#^P < 0.05 vs. Control.

MAP: Mean Arterial Pressure.

### Renal volume and function

**Figure 1:**
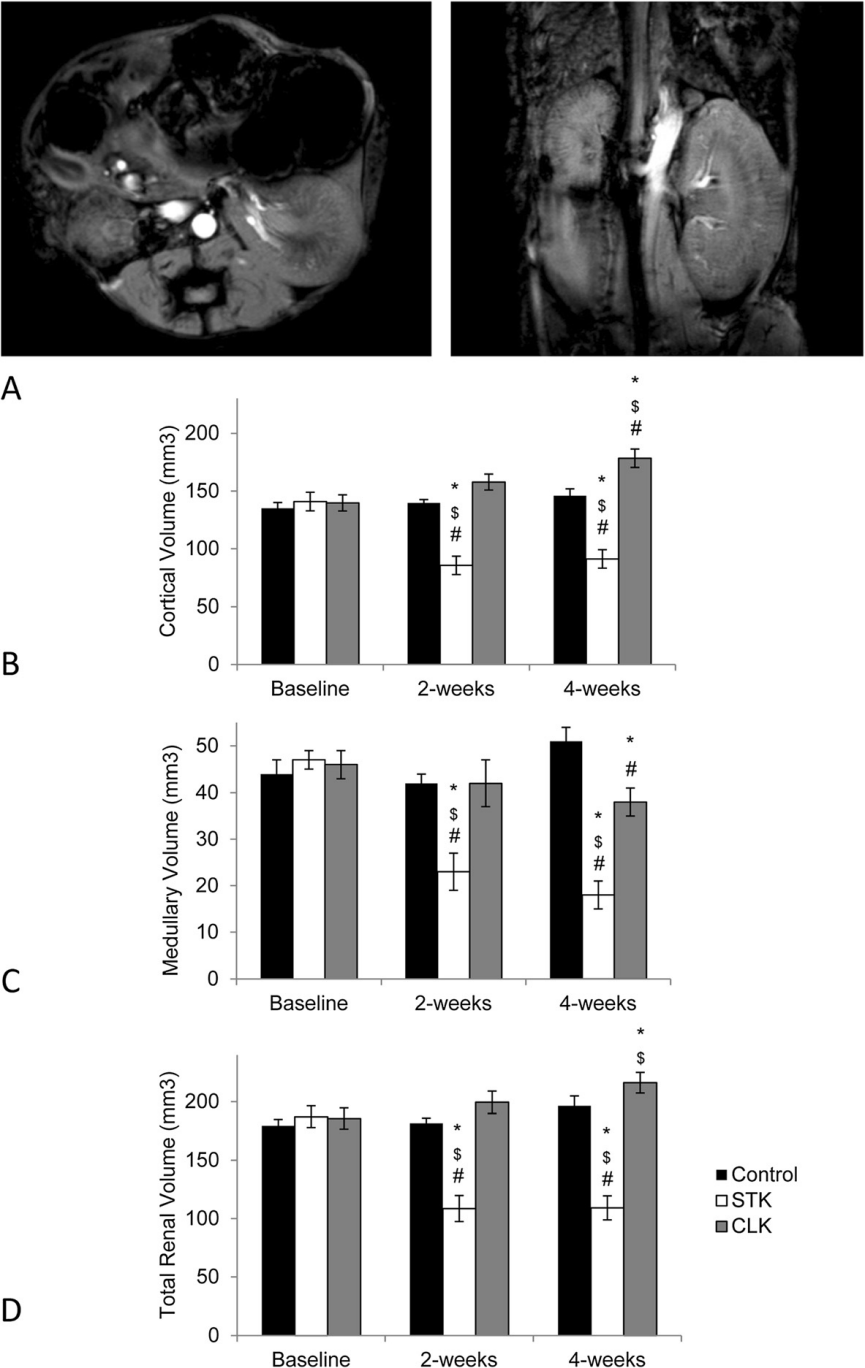
**Renal volumes.** Axial (left) and coronal (right) cross-sectional MR images of the atrophic stenotic kidney (STK) and hypertrophic contra-lateral kidney (CLK) 4 weeks after RAS induction **(A)**. Cortical **(B)**, medullary **(C)** and total renal volumes **(D)** before, 2 and 4 weeks after RAS induction or control periods. (*p < 0.05 vs. baseline RAS, ^$^p < 0.05 vs. baseline Control, ^#^p < 0.05 vs. Control).

Two weeks after inducing RAS, the STK cortical and medullary volumes significantly decreased, and by the fourth week were on average reduced to 39% and 61% of their baseline values, respectively (p < 0.001). At 2 weeks, the CLK cortical volume tended to increase (p = 0.06 vs. Baseline) and at 4-weeks was significantly elevated compared to both baseline and the control 4-weeks. Medullary volume remained unchanged during the first 2 weeks, but at 4 weeks was subsequently diminished compared to the control group (Figure 1).

STK blood flow markedly declined after RAS and remained lower than Control for the remainder of the study (p < 0.001). In the CLK, however, cortical blood flow was preserved, although CLK medullary blood flow decreased by the fourth week compared to the control. Control RBF remained unaltered at all time points.

Both medullary and cortical perfusions in the STK were impaired at two and four weeks (p < 0.001 vs. baselines and Control), whereas CLK cortical perfusion was not significantly changed, but showed strong tendency to decline during the entire 4 weeks of RAS.

In all the groups BOLD images, acquired using the conventional method, were highly affected by susceptibility artefacts, particularly in regions adjacent to the bowel.

**Figure 2:**
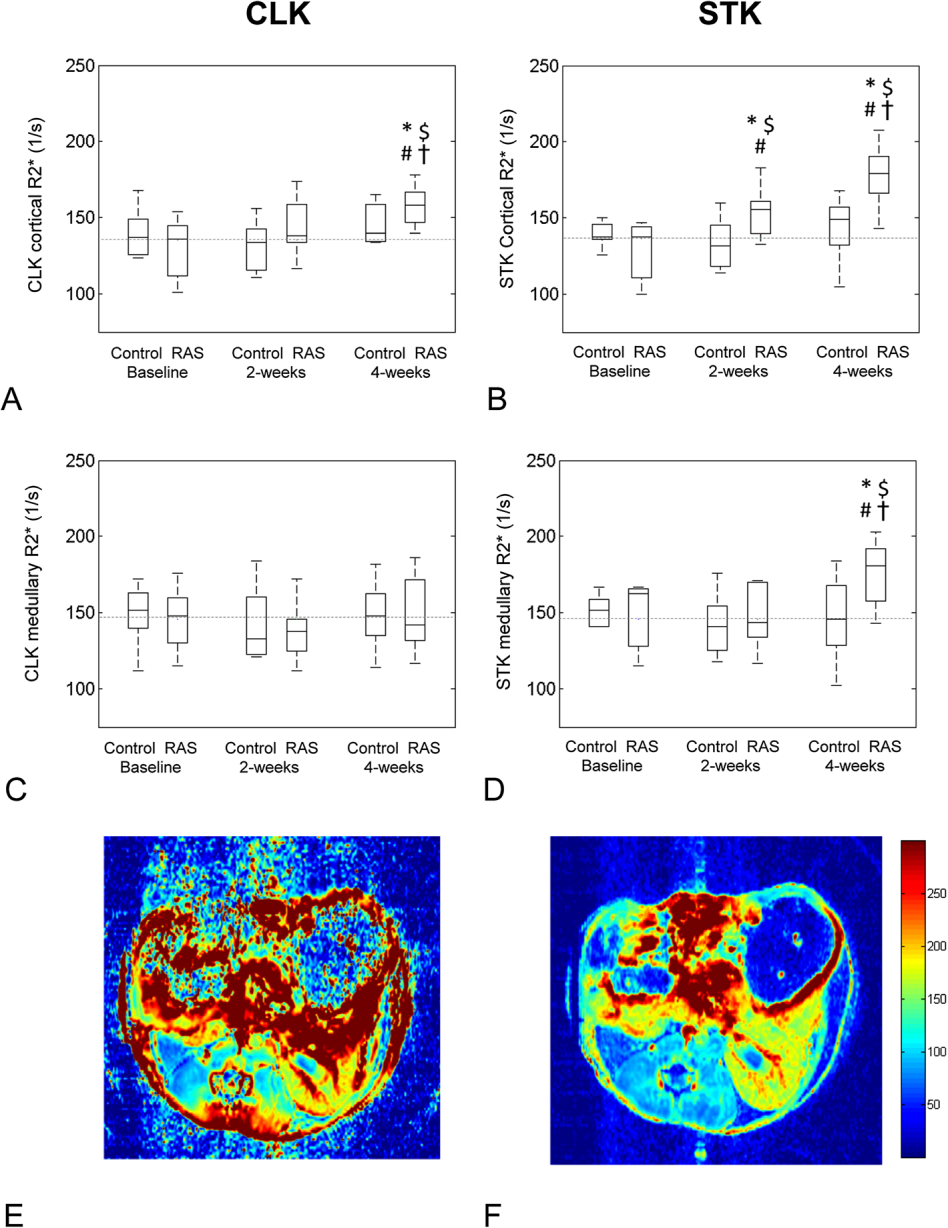
**Renal BOLD MRI.** Blood oxygen level-dependent (BOLD) MRI in the cortex and the medulla of the contra-lateral (left) and stenotic (right) kidneys, at baseline and 2 and 4 weeks later in RAS and control mice **(A-D)**. Dash-lines show the mean R_2_* in control animals over the entire course of study (*p < 0.05 vs. baseline RAS, ^$^p < 0.05 vs. baseline Control, ^#^p < 0.05 vs. control and ^†^p < 0.05 vs. 2-weeks RAS). BOLD maps acquired using the conventional **(E)** and the improved **(F)** BOLD techniques. Severe abdominal susceptibility artefact is evident in the conventional BOLD map.

In BOLD acquired with the improved method, cortical R_2_* in the STK significantly increased (p ≤ 0.02 vs. Baseline, and p = 0.007 vs. 2-weeks Control) at 2-weeks and further elevated at 4-weeks (p < 0.001 vs. Baselines and 2-weeks Control, p = 0.004 vs. 2-weeks RAS) (Figure 2). In contrast, an increase in STK medullary R_2_* reached statistical significance only at 4-weeks (p < 0.01 vs. Baselines and 4-weeks Control). In the CLK, at 4-weeks cortical oxygenation significantly diminished (p < 0.015 vs. Baselines and 4-weeks Control) although it was significantly higher than in STK. R_2_* values in the control group were unchanged.

### Cardiac volume and function

**Figure 3:**
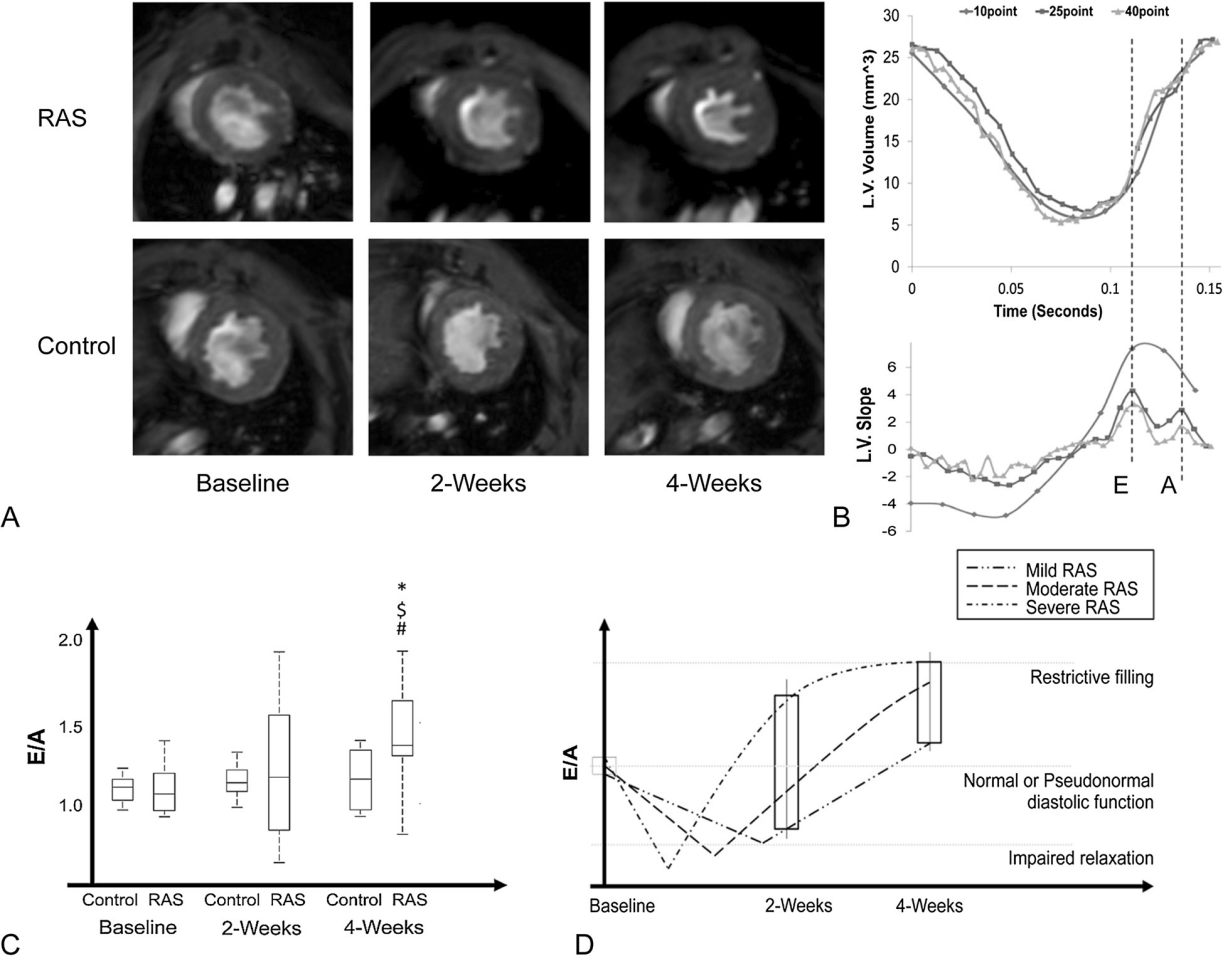
**Cardiac morphology and function.** MRI images of the mouse myocardium at baseline, 2 and 4 weeks after RAS (top) or control (bottom) **(A)**. Myocardial hypertrophy is evident 2 weeks after renal artery stenosis (RAS). LV volume-time (top) and its slope (bottom) as a measure of filling velocity **(B)**. The two peaks represent the early (E) and late (A) diastole phase. E/A box plot (*p < 0.05 vs. baseline RAS, ^$^p < 0.05 vs. baseline Control, ^#^p < 0.05 vs. 4-weeks Control) **(C)**. A schematic illustration of evolution of E/Ain animals with different levels of RAS severity, demonstrating broad diversity in left ventricular dysfunction during the second week after RAS and greater propensity toward restrictive filling phase at 4-weeks **(D)**.

In RAS stroke volume and cardiac output decreased at 2 weeks (Table 2), accompanied by myocardial hypertrophy (Figure 3A, p < 0.001 vs. Baselines and p = 0.005 vs. 2-weeks Control), while a decrease in LV end-diastolic volume was only significant compared to the 2-weeks Control. The control group showed a small increase in myocardial volume at 4 weeks, whereas ejection fraction showed no change in any group. At 2-weeks, E/A in RAS, but not in control, showed a wide range of values. At 4-weeks RAS E/A increased towards values corresponding to pseudo normal to restrictive filling phases (p < 0.05 vs. Baselines and 4-weeks Control) (Figure 3C).

**Table 2:** Heart rate and left ventricular cardiac parameters in renal artery stenosis (RAS) or control mice

	**Baseline**	**2 weeks**	**4 weeks**
Heart rate (beats per min)			
RAS	454 ± 17	429 ± 28	426 ± 19^#^
Control	470 ± 11	472 ± 3	482 ± 23
Left Ventricular Volume (mm^3^)			
RAS			
End Systole	8.4 ± 1.2	9.1 ± 1.5	8.7 ± 1.4
End Diastole	33.2 ± 1.4	30.3 ± 2.0^#^	29.7 ± 1.7^#^
Myocardial Volume	53.3 ± 3.7	70.7 ± 3.5*^$#^	76.6 ± 3.3*^$#^
Control			
End Systole	9.6 ± 1.3	11.2 ± 0.9	11.8 ± 1.3
End Diastole	34.3 ± 2.0	35.9 ± 1.2	36.8 ± 2.4
Myocardial Volume	51.5 ± 0.6	56.6 ± 2.2	61.0 ± 2.2*
Stroke Volume (mm^3^)			
RAS	24.7 ± 1.5	21.1 ± 0.9*^$#^	20.9 ± 1.3*^$#^
Control	24.7 ± 1.0	24.8 ± 1.0	24.9 ± 1.5
Ejection Fraction (%)			
RAS	74.7 ± 3.0	71.4 ± 3.2	71.3 ± 3.8
Control	72.5 ± 2.6	69.1 ± 1.9	71.4 ± 3.2
Cardiac Output (ml/min)			
RAS	11.2 ± 0.7	8.5 ± 0.9*^$#^	8.7 ± 0.7*^$#^
Control	11.6 ± 0.4	11.7 ± 0.4	12.7 ± 0.5
E/A Ratio			
RAS	1.09 ± 0.08	1.22 ± 0.12	1.40 ± 0.10*^$#^
Control	1.09 ± 0.04	1.14 ± 0.03	1.11 ± 0.05
Time to Systole/Diastole			
RAS	1.2 ± 0.2	1.3 ± 0.2	1.1 ± 0.1
Control	1.1 ± 0.1	1.1 ± 0.1	1.1 ± 0.1

*P < 0.05 vs. Baseline RAS, ^$^P < 0.05 vs. Baseline Control, ^#^P < 0.05 vs. Control.

The quality of images deteriorated as the number of reconstructed phases per cardiac cycle increased. End-diastolic, end-systolic, and stroke volumes, calculated from images reconstructed at 25 and 40 phases per cycle were slightly (7-11%) albeit not significantly, higher than those calculated from the set acquired with 10 phases per cycle (Figure 3B), while the variations in ejection fraction were negligible. LV volume-time curve analysis showed that (E) and (A) slopes were not reliably recognizable at 10, but were detectable in most of the 25 and in all curves with 40 time points per cardiac cycle.

**Figure 4:**
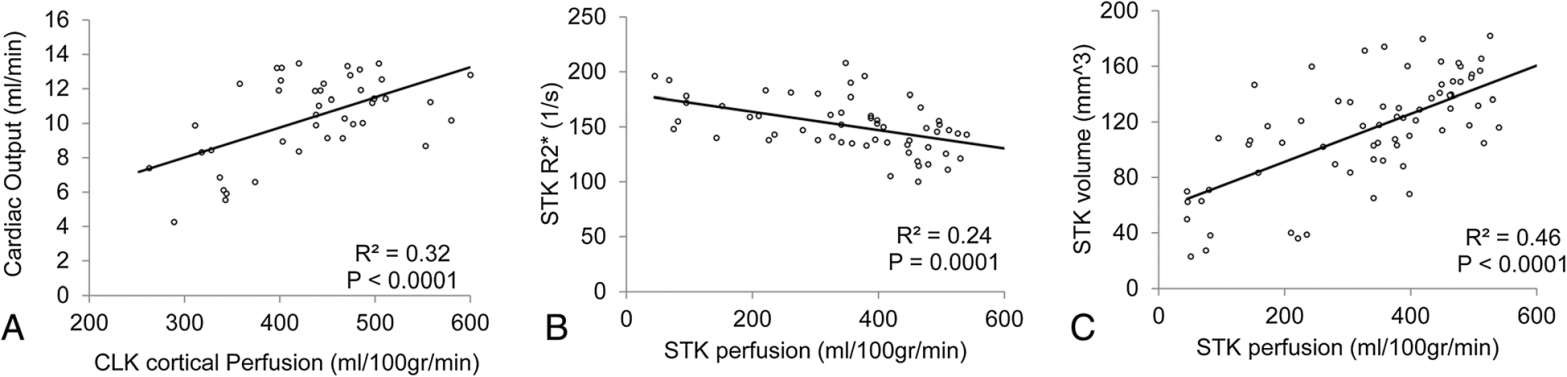
**Correlations between functional parameters.** Correlations between cardiac output and CLK cortical perfusion **(A)**, R_2_* and perfusion in the STK cortex **(B)** and volume and perfusion in the STK cortex **(C)**.

Linear regression (Figure 4) revealed a significant correlation between cardiac output and CLK cortical perfusion (R^2^ = 0.32, p < 0.0001). Additionally, in the STK cortex volume and oxygenation level correlated with its perfusion (R^2^ = 0.24 and R^2^ = 0.46, respectively, p ≤ 0.0001).

**Figure 5:**
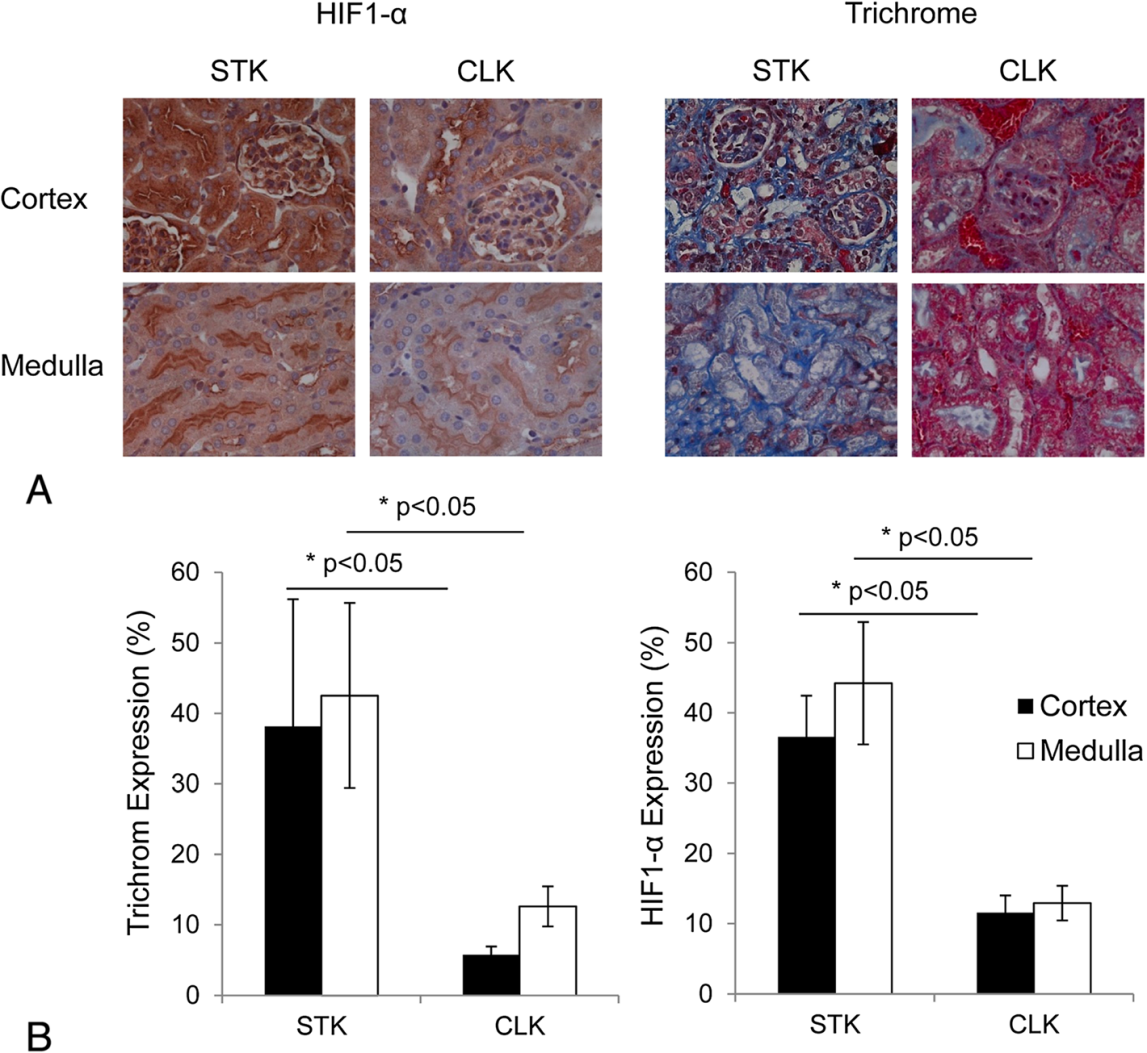
**Renal hypoxia and fibrosis.** Colorimetric images **(A)** and quantification **(B)** of HIF1-α expression (left) and trichrome (right) as measures of tissue hypoxia and fibrosis, respectively. HIF1-α and fibrosis expressions were significantly higher in the STK than CLK.

Renal staining for HIF1-α and trichrome, indices of tissue hypoxia and fibrosis, demonstrated significantly higher values in STK vs. CLK at the end of fourth week (Figure 5).

## Discussion

This study demonstrated the feasibility of using UHF-CMR to longitudinally derive quantitative measures of both renal and cardiac function and structure in mice with RAS. The sequential changes in renal and cardiac function were significant compared to matched controls, and corresponded to the pathophysiology of the disease.

Two weeks after unilateral RAS, MAP showed significant elevation and hemodynamic and morphological changes were evident. Main renal artery stenosis reduces the intra-renal perfusion pressure, which activates renin-angiotensin-aldosterone system (RAAS) [34]. RAAS activation is often followed by a chain of events, which eventually result in vascular remodelling and increased systemic vascular resistance [35]. Concurrently, RAS results in impaired RBF, decreased oxygen supply, and eventually hypoxia, in addition to STK atrophy and reduced function [4]. Within two weeks, pathophysiological indices of RAS in the STK included significant renal atrophy, decreased cortical and medullary perfusion and RBF, and development of cortical hypoxia, which correlated with cortical perfusion. Such correlation is plausible considering that impaired hemodynamics limits oxygen delivery, enhances oxidative stress and promotes hypoxia.

In the CLK, hypertrophy in the face of unchanged RBF slightly reduced cortical perfusion. In contrast to the STK, no correlation was found between cortical perfusion and volume, suggesting that the perfusion decline in CLK is regulated by factor(s) other than hypertrophy alone. Previous studies have demonstrated significantly higher renal vascular resistance in CLK of 2K, 1C rats as a result of elevated oxidative stress or reduced bioavailability of nitric oxide [36]. In addition, reduced cardiac output and the increased filtrate volume and swelling of the CLK tubules might contribute to regional decrease of perfusion values despite sustained RBF.

LV dysfunction driven by hypertension was also observed two weeks after RAS induction. Myocardial hypertrophy, initially an adaptive mechanism in response to the increased afterload, subsequently evolves into pathological cardiac remodelling, reducing stroke volume and cardiac output [37]. Our results indicated that myocardial mass significantly increased and the stroke volume decreased, mainly as a result of reduced LV end-diastolic volume. Cardiac output declined partly because of the reduced stroke volume and partly due to the lower heart rate. E/A at 2-weeks demonstrated substantial variability in the RAS group, ranging from impaired relaxation (low E/A) to restrictive filling phase (high E/A), indicating large variation in the rate of the progression and the extent of cardiac dysfunction among subjects.

By 4 weeks of RAS MAP remained elevated, STK RBF, perfusion and volume remained impaired, and hypoxia exacerbated. Conversely, the CLK cortex underwent further hypertrophy and a mild hypoxia, likely due to over-functioning as a compensatory mechanism in the face of slightly declined perfusion that was correlated to the cardiac output. Yet, R_2_* values in CLK were significantly lower than those in the STK. Histological markers of tissue hypoxia and fibrosis were greater in the STK compared to the CLK, confirming BOLD-MRI results and implying deterioration of the STK structure. In the heart, despite further hypertrophy of the myocardium, functional parameters at 4 weeks did not change any further. Nevertheless, high values of E/A became more common, suggesting further progression towards LV restrictive diastolic dysfunction, concordant with aggravated LVH.

These observations indicate that UHF-CMR allows a detailed characterization of the evolution of cardiac dysfunction following RAS. In large animal models and clinical studies, CMR is the reference standard for diagnosis of cardiac and renal dysfunction. Several studies have demonstrated the accuracy of this modality compared to ultrasound-based methods [38,39]. Nevertheless, in small animals, cardiac MRI faces some challenges, such as small size of the heart and its fast beating, which impose constrains on spatial and temporal resolutions. Although several recent studies successfully showed the feasibility of using clinical MR systems for cardiac imaging in mice [40,41], the achieved temporal resolutions remained sub-optimal and lower than typical values obtained with echocardiography and the minimal temporal resolution required to assess E/A. Our results as well as others’ [42] show that UHF-CMR affords such high resolutions.

In addition to cardiac imaging, UHF-CMR was found to be useful for functional renal MRI. Kidney perfusion measurement using ASL is rapidly gaining popularity due to its non-invasive nature. ASL relies on endogenous spin labelled plasma, which carries no physiological hazard, while an alternative perfusion measurement method, dynamic contrast-enhanced CMR, requires administration of potentially nephrotoxicity contrast-media, particularly in functionally compromised kidneys. To the best of our knowledge, this is the first study utilizing ASL with UHF in the kidney. However, previous studies in other organs have suggested that ASL benefits from high fields [43,44]. Prolonged T1 and remarkable signal-to-noise ratio improve the accuracy and sensitivity of this method, which are particularly important in RAS kidneys with impaired RBF and intrinsically hypoperfused medulla [45].

Yet, the high signal-to-noise ratio in UHF-CMR comes at the cost of amplified susceptibility artefact, which particularly affects BOLD images corresponding to later echoes with longer TE values [46]. Perfect shim in in-vivo imaging of the abdomen is difficult, and adjacency of this organ to the bowel makes this even more challenging. In our experiments, R_2_* values calculated from conventional BOLD maps were highly influenced by the susceptibility artefact. Therefore, improved BOLD was employed under the assumption that reducing the thickness and utilizing isotropic voxels would reduce the phase dispersion caused by the macroscopic external field inhomogeneity, while the microscopic field inhomogeneity caused by deoxyhemoglobin would remain unaffected [30]. Theoretically, the new approach is more sensitive to blood oxygenation, as tissue contrast is solely determined by deoxyhemoglobin concentration rather than modulated heterogeneous tissue susceptibility. This may also account for the lower R_2_* values generally observed in the improved compared to the conventional BOLD. Nevertheless, because R_2_* values are scaled by magnetic field strength, and the values in this study are considerably higher than those acquired using clinical MR systems (1.5 and 3 T) [47], or even UHF systems at lower fields [48].

Our study involved several limitations. Mice reach sexual maturity between 5-8 weeks of age. Body weight, renal, and myocardial volumes in the control group showed slight growth during the course the study. Physiological parameters in the RAS group were compared not only to their baseline, but also to those in the control group, yet we cannot rule out the influence of age on the longitudinal assessments. Furthermore, despite using a similar cuff, the severity of RAS and the rate of progression of the disease might not have been identical in all animals, due to intrinsic variability. This might have introduced variations in some of the physiological markers and influenced their statistical significance. In addition, renal cortical and medullary volumes were quantified from 3D images with relatively short echo time, and especially in the STK were at times difficult to differentiate. Similarly, ASL images were also less clear in STKs. In the heart, dark regions caused by ventricular blood vortex, particularly in slices in the vicinity of the mitral and aortic valves, could have been mistaken for myocardium and introduced some error into myocardial mass quantifications. Nevertheless, our measurements were consistent.

## Conclusions

Despite potential limitations, UHF-CMR detected measurable changes in functional and morphological biomarkers of cardio-renal dysfunction, and afforded a comprehensive depiction of longitudinal progression of the disease. Importantly, UHF-MRI, non-invasively, provided useful information on a broad range of markers of cardiac and renal dysfunction at higher spatial and temporal resolution than typically achieved using alternative modalities. These observations support the application and further development of UHF-MRI for studies of renal and cardiac function.

## Abbreviations

2 K: 1C, 2-kidney, 1-clip; STK: Stenotic kidney; CLK: Contra-lateral kidney; BOLD: Blood oxygen-level dependent; CKD: Chronic kidney disease; CVD: Cardiovascular disease; E/A: Early to late ventricular filling velocity ratio; LV: Left ventricular; LVH: Left ventricular hypotrophy; RBF: Renal blood flow; RAS: Renal artery stenosis; UHF-MRI: Ultra-high field magnetic resonance imaging.

## Competing interests

The authors declare that they have no competing interests.

## Authors’ contributions

BE made substantial contributions to the conception, design, acquisition of data, analysis and interpretation of data, drafting the manuscript and has given final approval of the version to be published. JAC has made substantial contributions to the analysis and interpretation of data, drafting the manuscript and has given final approval of the version to be published. BEK has made substantial contributions to design, surgical procedures, acquisition of data, drafting the manuscript, revising it critically, and has given final approval of the version to be published. SIM has made substantial contributions to the conception, design, acquisition of imaging data, analysis and interpretation of data, revising it critically for important intellectual content and has given final approval of the version to be published. JPG made substantial contributions to the conception, design, interpretation of physiological data, revising it critically for important intellectual content and has given final approval of the version to be published. LOL made substantial contributions to the conception, design, interpretation of data, drafting the manuscript and revising it critically for important intellectual content and have given final approval of the version to be published. All authors read and approved the final manuscript.
